# Frontiers in broiler chicken welfare: adopting early detection of intestinal integrity loss in broiler welfare assessment protocols

**DOI:** 10.3389/fvets.2025.1593737

**Published:** 2025-05-06

**Authors:** Ingrid C. de Jong, Soumya Kanti Kar, Bernd Kaspers

**Affiliations:** ^1^Wageningen Livestock Research, Wageningen University and Research, Wageningen, Netherlands; ^2^Department for Veterinary Sciences, LMU Munich, Munich, Germany

**Keywords:** broiler, sickness behavior, coccidiosis, emotional state, gut-brain axis, intestinal integrity, welfare

## Abstract

Current broiler welfare assessment protocols include indicators of impaired intestinal health, but these are non-specific. Loss of intestinal integrity is considered to be a major welfare consequence for broilers but the absence of specific, early indicators in welfare assessment protocols hampers early detection and treatment. Coccidiosis is one of the major threats to intestinal integrity in broiler chickens and taken as an example. We propose the development of specific biomarkers for detecting early onset of intestinal health deterioration. The genotype and external phenotype of organisms are linked by so-called internal phenotypes which are influenced by environmental conditions. We review the impact of coccidiosis on external and internal phenotypes in chickens. The change from the “homeostatic immune response” toward an inflammatory response to control infections is reflected in the change in feeling of comfort to the feeling of discomfort in broilers, in which the gut-brain axis likely plays a crucial role. With this change, a negative emotional state develops. Two routes of developing biomarkers are proposed that are interconnected. The first route is by enabling ~omics techniques for predominantly invasive biomarkers related to the internal phenotype of the broiler chickens during infection. The second approach involves using sensors and automated systems to monitor behavior, vocalizations, and fecal appearance for early disease detection at flock level. By linking these external indicators to invasive biomarkers, we can develop disease-specific biomarkers that enhance early diagnosis with precision and could add significant value to welfare assessment protocols. Research in this area should be encouraged.

## 1 Introduction

Intestinal health in broiler chickens is both critical for absorption of nutrients and a prerequisite for broiler welfare. Antibiotic treatments have long been applied to prevent or treat intestinal infections, but concerns around antibiotic resistance and the implications for human and animal health resulted in moves toward antibiotic-free broiler production (1). However, as a consequence, intestinal health issues became more apparent (2), impairing broiler welfare and performance.

**Figure 1:**
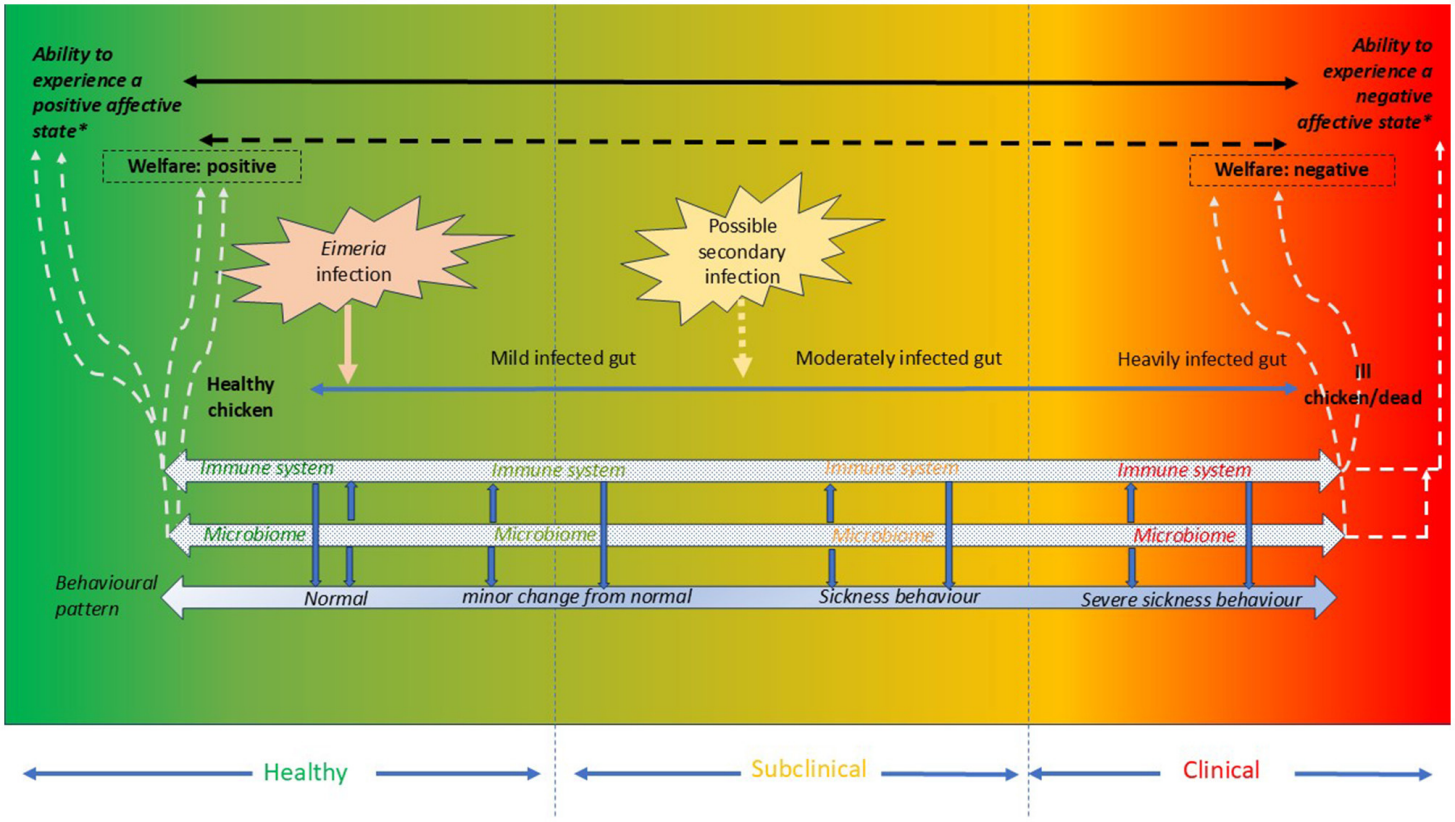
Schematic representation illustrating the impact of an *Eimeria* infection on chicken health and welfare. Depending on the pathogenicity of the infection, three stages are shown–the birds pick up the infection in a healthy state (green) and can develop a subclinical (yellow-orange) and clinical (red) form of the disease if left untreated. The solid blue arrow shows the progression of the infection from a mildly infected gut to a heavily infected gut. Within the gut, two important components, the host immune system and the resident microbiome, are shown in a spectrum ranging from effective (green) through compromised (yellow-orange) to ineffective (red). The host immune system, the gut microbiome and/or the interactions between the host (immune system) and the microbiome can produce biomolecules as signaling substances that induce behavioral (solid blue arrow) changes and (may) change the broilers' affective state and welfare status. In each of the corresponding sections, different behavioral patterns can be seen: normal to minor behavior changes in healthy birds; minor changes from normal behavior to sickness related behavior changes in birds with subclinical infection; sick to severe sickness related behavior in birds with a clinical form of infection. In a healthy state, welfare is not compromised by *Eimeria* infection, and broilers have the ability to experience a positive emotional state (if the situation permits, so other requirements in e.g., environment are also met) while during the subclinical and clinical stage of infection a negative emotional state (may) predominate(s).

Coccidiosis is the leading cause of loss of intestinal integrity in broiler chickens, surpassing other contributing factors such as bacteria, viruses, other parasites, and nutritional imbalances (3). Therefore, this perspective paper will focus on coccidiosis as a primary example of intestinal health challenges in broilers. Coccidiosis, caused by the pathogenic *Eimeria* parasite, starts when chickens ingest sporulated oocysts present in their environment. Replication of the parasite takes place within the intestinal epithelial cells and parasite numbers increase rapidly within a few days resulting in shedding a large number of oocysts. The host will develop an adaptive immune response to the challenge, but until this response is fully developed there will be a non-specific innate immune response including excessive secretion of mucin. The innate immune response has high energy costs for the host and during infection the intestinal microbiome is disrupted, favoring replication of pathogenic bacteria and predisposing to secondary infections (3) (Figure 1). The extent of damage to the intestinal tract depends on the *Eimeria* species, the initial challenge dose, the cells that it replicates within and the coccidiosis control strategy. One of the several commercially available control strategies involve using live attenuated *Eimeria* based vaccines. For these vaccines to be successful the parasites would be required to complete their lifecycle, inherently causing some degree of epithelial damage and subsequent inflammation. Being that the vaccines must elicit an immune response to be effective, it is conceivable that this immune status change itself could be expected to have some potential negative impact on welfare.

Health, thus absence of disease, is one of the physical domains in the five domains model of animal welfare, with a clear link to the mental domain (4). With impaired health, animals may be in a negative affective state, while good health facilitates positive emotional states (4, 5). Therefore, indicators of a healthy or diseased state are included in welfare assessment protocols. According to the recent EFSA opinion on the welfare of broiler chickens on-farm (6), one of the identified welfare consequences for broiler chickens is “gastro-enteric disorders.” The welfare consequence has been defined as “*The animal experiences negative affective states such as discomfort, pain and/or distress due to impaired function of the gastrointestinal tract resulting from, for example nutritional deficiency and infectious, parasitic or toxigenic agents,”* and was identified in all broiler production systems and breeds (6). Existing indicators of impaired intestinal health welfare assessment protocols are often non-specific and relate to a severe state of disease. E.g., the Welfare Quality protocol includes “plumage cleanliness,” “footpad dermatitis,” and “mortality” (7). Another assessment protocol includes “sick,” “terminally ill,” “dirty,” “small,” and “dead” (8). EFSA (6) listed “plumage/body cleanliness,” “footpad dermatitis,” “cloacal temperature,” “lethargy,” “impaired growth rate,” and “mortality” as possible indicators of gastro-intestinal disorders. A recent analysis of scientific literature related to welfare assessment under commercial conditions did not reveal other indicators in broiler chickens (9).

The listed welfare indicators, while important for assessing broiler welfare and acknowledging health, are not specific to intestinal health and may overlook subclinical disease. Early or subclinical intestinal disease may however be painful, cause malaise, and could impact welfare by hindering normal behavior and positive emotional states. Early detection encourages prompt intervention, preventing more serious consequences.

The connection between an organism's genotype and its external phenotype is mediated through several intermediary layers of so-called internal phenotypes (10). These layers, including the transcriptome, proteome, metabolome, and microbiome, represent key biochemical aspects of the central dogma of life. The transcriptome captures the direct effects of environmental factors on gene expression, while the proteome reflects the translation of these gene expressions into proteins. The metabolome represents complex profiles of metabolites, and the microbiome, particularly the gut microbiota, is influenced by both the host genome and environmental factors such as diet and pathogens. These internal phenotypic layers are interconnected and their combined profiles determine the external phenotype (11). Despite the known quantitative effects of the environment on external traits, the specific impacts on internal phenotypes are not yet fully understood. However, the use of ~omics technologies, each capturing distinct layers of internal phenotypes, plays a crucial role in identifying biomarkers that are key to understand and manage gastrointestinal health and diseases (12–14).

This perspective paper proposes developing specific biomarkers for early detection of intestinal health deterioration in chickens. We review coccidiosis's impact on external and internal phenotypes, particularly internal phenotypic layers. Developing such biomarkers could enable timely interventions helping to mitigate disease effects, enhance welfare, and reduce reliance on reactive treatments. Integrating these biomarkers into welfare assessments promotes proactive, sustainable poultry production and could help evaluate control strategies which impacts welfare, e.g., the innate immune response following vaccinations might have an underestimated impact on welfare. Figure 1 outlines the interrelationships between loss of intestinal integrity and broiler welfare that will be briefly discussed first, after which we will propose routes of detecting and developing promising biomarkers of impaired intestinal health.

## 2 The intricate interactions of intestinal barrier function components form the gut-brain axis and the gut-associated immune response

**Figure 2:**
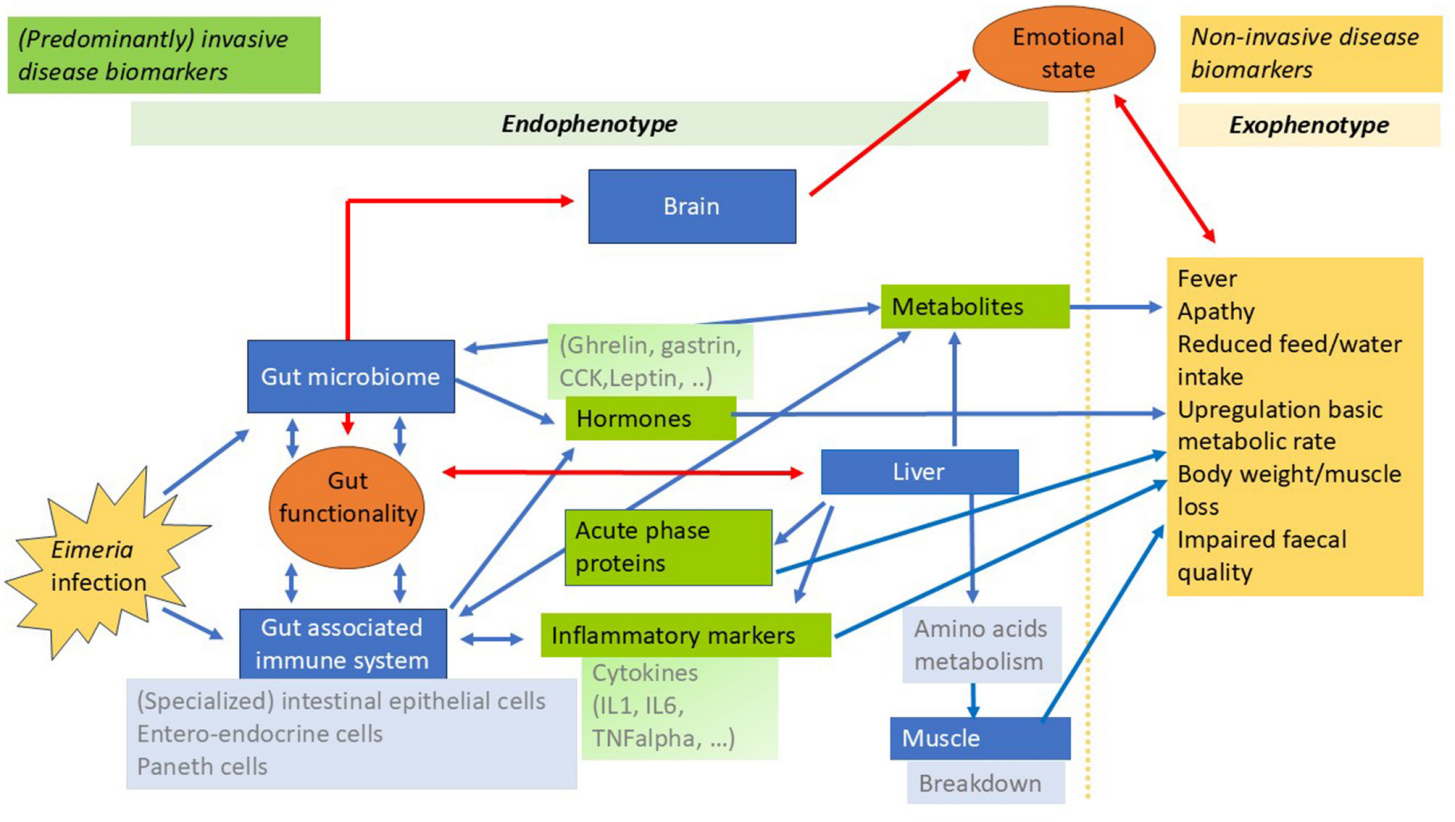
Snapshot and a schematic overview of changes due to the gut associated inflammatory response, leading to sickness behavior and impaired broiler welfare, with *Eimeria* infection as an example. The blue boxes indicate the tissue and organs, blue arrows indicate their interactions. Red arrows denote the gut-brain and gut-liver axis. The interactions among the molecular or cellular signatures of the endophenotype (indicated by the light green bar in the upper part of the figure) influence the exophenotype (indicated by the light orange bar in the upper part of the figure). Gut functionality and emotional state (orange circles) are the consequence of the interactions as shown by the arrows in the figure. In green boxes potential (predominantly) invasive disease biomarkers and in the orange box the potential non-invasive biomarkers are shown.

Figure 2 presents a schematic overview of elements of the intestinal barrier function, the gut associated immune response and their complex interaction during infection, which will be discussed here. The complex intestinal barrier function protects the birds from harmful infectious agents while still enabling absorption of nutrients. The intestinal barrier function components include the intestinal epithelium, the mucus layer(s), the microbiota, immune elements and biochemical elements. Disruption of this intestinal barrier function, often referred to as “leaky gut,” can be caused by consumption of contaminated feedstuff containing mycotoxins (15) and other toxins, poorly digested dietary protein (16) and pathogens such as *Eimeria* (17–19).

Cells of the immune system are an integral part of the mucosal tissue of the gut. Resident cells of the myeloid linage, including macrophages and dendritic cells, play an important role in maintaining gut integrity. They communicate with other immune cells, epithelial and stroma cells as well as the gut associated neuronal network through cell-cell contact and soluble mediators, called cytokines and hormones. In addition, epithelial cells and immune cells interact in a bidirectional way with members of the microbiota (20). This communication supports development of the gut immune system and maintenance of gut integrity and barrier function, while preventing gut inflammation (21). Thus, in the absence of pathogenic challenges the gut immune system receives cues from the microbiota and the epithelial cells which induce an anti-inflammatory response pattern by tissue resident macrophages, a phenotype referred to as M2 macrophages (22). Invasion of the gut by viral, bacterial, or parasitic pathogens shifts this “homeostatic immune response” toward an inflammatory response to control infection. Epithelial cells and resident macrophages of the lamina propria are the first cells to detect pathogens and to activate defense mechanisms (23). This includes a change in the macrophage function toward an M1 phenotype characterized by the secretion of inflammatory cytokines and chemokines (Figure 2). The best characterized factors of this response are the cytokines interleukin-1 (IL-1), IL-6, IL-8, and tumor-necrosis factor-α (24). They orchestrate the attraction of monocytes and their differentiation to inflammatory macrophages and the immigration of heterophils (25), the predominant form of granulocytes in chickens. The resulting morphological pictures of a massive increase in cell numbers in the lamina propria is described as inflammation by histo-pathologists. The resulting cascade of events aims at eliminating the pathogen which is often associated with a disruption of the epithelial barrier and further amplification of the inflammatory response (26). During an *Eimeria* infection there is rapid infiltration of the mucosal tissue by heterophils and the activation of macrophages and T-cells which results in the secretion of inflammatory cytokines (27). These early responses do not clear the infection but lead to tissue damage and systemic responses. Frequently, secondary infections are a consequence of *Eimeria* induced damage to the gut barrier (28) exacerbating the inflammatory response and sickness behavior.

When produced in sufficient amounts, inflammatory cytokines may induce a systemic response called acute-phase-response characterized by changes in liver metabolism and behavior. In the liver, acute-phase-proteins (APP) are synthesized in large amounts leading to an up to 1,000-fold increase in plasma concentrations (29). Quantification of APPs is a standard procedure in human medicine as an indicator of the severity of inflammation. In Chickens however (30), due to the requirement of blood sampling and subsequent analysis, their quantification as inflammatory markers is impractical. The production of large amounts of acute-phase-proteins leads to a shift in the requirement of essential amino-acids, which will lead to muscle breakdown (31). Behavioral changes result from the effects of inflammatory cytokines on circumventricular organs in the brain and include an increase in body temperature [fever (32, 33)], inappetence, somnolence and apathy, called sickness behavior. Moreover, cytokine mediated stimulation of hypothalamic nuclei activates the hypothalamic-pituitary-adrenal axis and the release of corticosterone from the adrenal gland which has potent immuno-suppressive effects (Figure 2).

A particular challenge for the immune system is the return to homeostatic conditions and restoration of gut integrity. Regulatory T-lymphocytes (Treg) are particularly important in this context. Currently, Treg cells and their cytokine repertoire are not well understood in chickens (34). They secrete a range of cytokines and chemokines which exert anti-inflammatory effect, best known are IL-10 and TGF-β. Other cytokines and chemokines regulate proliferation and differentiation of gut epithelial cells and help to strengthen the barrier.

The intestinal barrier components also play an important role in the gut-brain axis (Figure 2). The intestinal epithelium interacts with the brain via signaling molecules that traverse the intestinal epithelium and the circulatory system and cross the blood–brain barrier. While the mucus layer does not communicate directly with the brain, it forms a barrier that helps to maintain a healthy gut microbiota, which in turn communicates with the brain through the production of neurotransmitters and other metabolites. Immune cells in the gut produce cytokines and other signaling molecules that can influence brain function either directly or indirectly through interactions with the gut microbiota. Various molecules secreted into the lumen influence the gut microbiota and immune responses, which in turn influence the gut-brain axis. In summary, during bacterial colonization or *Eimeria* challenge, metabolites and/or biochemical elements are secreted into the gut lumen, which are then absorbed into the blood and transported to the brain. This influences hormone secretion via the gut-brain axis. Overall, the gut-brain axis involves complicated interactions between the gut, the nervous system, the endocrine system, the immune system and the gut microbiota. A disturbance of the gut-brain axis leads to a malfunction of the intestinal barrier and vice versa risking uncontrolled immunological reactions that can trigger low-grade inflammation of the mucous membranes and the brain, the first step toward triggering more permanent intestinal diseases. The gut-brain axis allows the brain to influence intestinal activity, including the activity of functional immune effector cells, and the gut to influence mood, behavior, cognition, and thus broiler welfare. Behavioral changes can relate to gut barrier dysfunction, as shown with feather pecking in laying hens (8). Studies underscore this, focusing on microbiota-mediated alteration of central serotonergic and dopaminergic systems (8) and the glutamatergic nervous system (35) in feather pecking. The maintenance of homeostatic state, as observed in clinically healthy birds with normal behavior, depends on the function of the intestinal epithelia and the resident microbiome. This relationship is mediated by biochemical elements that communicate with the brain via the enteric nervous system, the vagus nerve and the bloodstream, which transports effector molecules resulting from host-microbe interactions in the gut.

Thus, the change from the “homeostatic immune response” toward an inflammatory response is reflected in the change from comfort to discomfort in avians and mammals, in which the gut-brain axis likely plays a crucial role. With this change, welfare is impaired and a negative emotional state develops during intestinal infection. The massive increase in cells in the lamina propria, described as inflammation by histo-pathologists, could be seen as the first phase of the discomfort of being ill.

## 3 Discussion

As outlined above, when gut health is increasingly compromised, through the gut-brain axis multiple complex processes are activated that directly or indirectly affect a broiler chicken's emotional state and behavior. In current welfare assessment protocols indirect indicators of impaired intestinal health or indicators related to a very advanced stage of disease are included (7, 8). While, with respect to guaranteeing animal welfare, and to enable early intervention by farmers, biomarkers indicative of earlier stages of intestinal disease, and specifically highly prevalent diseases, are needed. This area clearly deserves further research, but there are promising areas of development.

It is widely assumed that internal phenotypes are interconnected and collectively shape the external phenotype (10, 35). However, many of these assumptions lack robust evidence and often oversimplify the complex processes influencing external phenotypes. This gap in understanding partly accounts for the challenges in bridging the knowledge divide between genotype and external phenotype. Recent efforts by researchers to elucidate connections between internal phenotypic layers have shown that integrating multi-scale quantitative, multi-omics data using a regression-based approach holds significant promise (11). Such multi-omics data analyses approach has provided provisional insights into the interactions among the internal phenotypes or layers, including local (e.g., intestinal) and systemic components. Results generated from these integrated approaches can form hypothesis-driven research aimed at identifying causal relationships across biological scales, potentially reducing the knowledge gap between internal and external phenotypes. This general methodology can be adapted to various datasets and perturbations, including pathogens such as *Eimeria*. Beside the multi-omics data integration approach, “high-level” data integration of singular-omics representing one layer of the internal phenotype could be used to identify biomarkers. Omics technologies offer valuable insights into the complex mechanisms underlying the onset of coccidiosis by enabling the identification of specific biomarkers, as indicated in the left section of Figure 2 with the predominantly invasive biomarkers. These are more suitable for experimental conditions. However, by correlating these biomarkers with behavioral changes in chickens affected by coccidiosis, it is possible to develop more targeted preventive measures and improve disease management strategies in poultry.

Novel technologies offer opportunities for fine tuning non-invasive biomarkers of disease as indicated in the right section of Figure 2. Farmers and veterinarians use flock behavior, vocalizations and/or odor as indicators of flock health in their daily management (36). It is however difficult to detect very early stages of disease by visual inspections alone. Automated detection of broiler behavior, flock distribution and movement are promising, non-invasive tools for early detection of behavioral changes. It has been shown that optical flow patterns of flock activity can be used to predict *Campylobacter* infection of a flock in a very early stage, even earlier than by conventional microbiological methods (37). This suggests that certain deviations in behavior can be specific to certain infections, although further study is required to what extent such changes can also be linked to other infections. Behavioral changes have been proposed for early disease detection in dairy cattle (36), but it remains to be studied to what extent specific behaviors can be indicative of early intestinal challenge in broilers, and how these can be practically assessed. From a technical perspective it has already been demonstrated that individual behaviors and postures as well as group-level changes in activity and distribution can be detected in chickens (38, 39). Similarly, it has been shown that it is possible to detect abnormalities in fecal appearance by image analysis (40), early detection of respiratory diseases by sound analysis (41, 42) and a combination of visual and thermal images to detect fever in chickens (43). Although many of these automated detection technologies need further development for application in practice, these present opportunities for earlier and more specific disease detection in future welfare assessment schemes.

Despite significant progress in developing biomarkers for assessing intestinal health in broilers, the integration of these tools into standard welfare assessment protocols remains a future goal. The current biomarkers show promise in detecting early indicators of compromised intestinal integrity, which could allow for timely interventions and improved welfare outcomes. However, several challenges need to be addressed before these biomarkers can be reliably included in routine welfare assessments. These include validating biomarkers across diverse flock conditions, ensuring non- or minimally-invasive sampling, and establishing standardized thresholds. Additionally, further research is required to link specific biomarker responses to particular welfare-relevant outcomes, which will improve the specificity and relevance of these tools in practical applications. Overcoming these barriers will take time, yet the potential benefits underscore the importance of continued research and development in this area.

## Data Availability

The original contributions presented in the study are included in the article/supplementary material, further inquiries can be directed to the corresponding authors.
